# Prevalence of depression and its associated factors among primary caregivers of patients with severe mental illness in southwest, Ethiopia

**DOI:** 10.1186/s12888-017-1249-7

**Published:** 2017-03-09

**Authors:** Habtamu Derajew, Daniel Tolessa, Garumma Tolu Feyissa, Fikir Addisu, Matiwos Soboka

**Affiliations:** 1 Emanuel Mental Specialized Hospital, Addis Ababa, Ethiopia; 2 Department of Psychiatry, College of Public Health and Medical Sciences, Jimma University, Jimma, Ethiopia; 3 Department of Health Education and Behavioral Science, College of Public Health and Medical Sciences, Jimma University, Jimma, Ethiopia; 4 Centre for International Health, Ludwig Maxmillians University, Munich, Germany; 5 The Joanna Briggs Institute, The University of Adelaide, Adelaide, Australia

**Keywords:** Depression, Primary caregivers, Severe mental illness, Ethiopia

## Abstract

**Background:**

Depression is a serious mental illness that affects patients’ treatment outcome and caregiver’s day to day life. The prevalence of depression among caregivers of patients with severe mental illness is greater than the general population. Little is known about depression among primary caregivers of patients with severe mental illness in Ethiopia. This study is aimed at assessing prevalence of depression and associated factors among primary caregivers of patients with mental illness.

**Methods:**

A cross-sectional study was conducted among primary caregivers of patients with severe mental illness in Jimma University Teaching Hospital. Patient health questionnaire (PHQ-9) was used to assess depression. A multidimensional scale of perceived social support (MSPSS) was used to assess perceived social support; Cut down, Annoyed, Guilty, Eye opener (CAGE) scale was used to assess alcohol use disorder. After conducting descriptive analyses, logistic regression analysis was finally used for bivariate and multivariable analysis.

**Result:**

The overall prevalence of depression among primary caregivers of patients with mental illness was 12 (19%). Out of those caregivers with depressions, 11.3, 3.5 and 4.2% had moderate, moderately severe and severe types of depression respectively. The prevalence of depression among female primary caregivers was 25% (*n* = 40). Being single (aOR 2.62, 95% CI = 1.07, 6.41), giving care more than six hours per day (aOR 3.75, 95% CI = 1.51, 9.33) and caring for a patient who had more than once episodes of suicidal attempts (aOR 1.48, 95% CI = 1.07, 3.42) were positively associated with depression among caregivers of patients with mental illness.

**Conclusion:**

We found that the prevalence of depression among primary caregivers was high. Depression among caregivers was associated with giving care more than six hours per day and caring for a patient who had two or more episodes of suicidal attempts. The prevalence of depression among female caregivers was higher than that of the male caregivers. Therefore, special focus should be given to primary caregivers spending long hours for providing care, those with low perceived social support; caregivers of patients with suicidal ideation and female caregivers.

## Background

Across the world, around 450 million people suffer from mental or behavioral disorders. One person in every four will be affected by a mental illness at some stage of life [1]. Mental illnesses affect peoples of all ages, educational levels, economic statuses and cultural backgrounds. The effect of these illnesses on the caregivers of people with mental illness is substantial [2]. Around 150 million persons in the world suffer from depression at any one point in time [3]. Depression is a state of low mood and aversion to activity that can affect a person’s thoughts, behavior, feelings and sense of well-being. People with depressed mood can experience feeling of sadness, lack of interest, hopelessness, helplessness, worthlessness, guilty feeling, irritability. They may also experience suicidal ideation, sleep disturbance and loss of appetite [4]. Depression accounts for 4.3% of the global burden of disease and is among the largest single causes of disability worldwide particularly for women [5]. Based on the global burden of disease 2010, depressive disorders were the second leading cause of Years Lived with Disability (YLDs) accounting for 8.2% (5.9–10.8%) of global YLDs [6]. Patients with major depression have a 40–60% greater chance of dying prematurely than the general population [5] because depression is associated with heart diseases, cancer and diabetes mellitus [5–7]. Caregivers of patients with neurological illnesses experience high levels of psychological distress and depression; increased rates of physical illnesses and personal, financial, family, and other social problems [8]. Depression among caregivers of patients with mental illness has been estimated to be more than two times higher than the general population [9, 10]. The burden of depression among caregivers of patients with mental illness includes disturbance in routine activities, social interaction, leisure activities and jobs. Mental illness is associated with increased physical morbidity [11–13] and reduced quality of life among caregivers [14].

Depression among caregivers of patients with mental illness has been positively associated with younger age of the care giver and lower levels of caregivers’ education [10, 15], being assaulted by patient [16], caregivers perceived stigma [10, 11], increased number of hours spent providing care (per week), older age of the caregiver and duration of care giving [11], being non-religious [17] and having a poor social support system [18–20]. Similarly, it has been found that increased duration of mental illness, increased number of hospitalizations are positive predictors of the experience of depression among caregivers of mentally ill patients [15]. Depression further depletes the caregiver’s own resources, increasing care costs for both the caregiver and the care recipient. When caregivers are, depressed and overwhelmed by their care giving tasks, they are more likely to suffer burnout and may make the often-agonizing decision to place their loved ones in a nursing home. Caregivers often focus on the needs of those they care for but neglect their own health needs [7]. So, depression among caregivers is a serious problem [7]. However, there are little data known about depression among primary caregivers of patients with mental illness in sub-Sahara African countries including Ethiopia. Therefore, this study is aimed at assessing depression among primary caregivers of patients with mental illness in Southwest Ethiopia.

## Methods

### Study area and period

This study was conducted in the Psychiatry clinic at Jimma University Teaching Hospital (JUTH), which is in Oromia Regional state in Ethiopia. JUTH is one of the oldest public hospitals in Ethiopia. The hospital provides specialized clinical services by its more than 9 clinical departments and is supported by specialized diagnostic and pharmaceutical units. Psychiatry is one of these clinical departments. The psychiatry unit of JUTH is one of the oldest psychiatry clinics in Ethiopia following Emanuel Mental Specialized Hospital. The Psychiatry clinic of JUTH was established in 1988. Daily, more than 60 patients get service at outpatient department of the psychiatry clinic. The data were collected from primary caregivers of patients with severe mental illness who came to JUTH to collect medications for the patients or who came with patients during psychiatry clinic follow up in October 1-30, 2013.

### Study design

A facility based cross-sectional survey was conducted among caregivers receiving services at the outpatient department of the psychiatry clinic in JUTH.

### Sample size assumption and sampling procedure

The sample size was determined by using single population proportion formula assuming the rate of depressive disorders among care givers of patients diagnosed with schizophrenia to be 23.3%, as it was reported by a study done in Buraydah Saudi Arabiya (11) with 5% marginal error (d) and 95% confidence interval of certainty (alpha = 0.05).

$$ n=\frac{1.96^2 p\left(1- p\right)}{d^2} $$

Based on this assumption, the total sample size for the study was 284.

Primary caregivers who were more than 18 years were included in the study. Primary caregivers who were away from patients at least for a month in the past three months were excluded. Clinicians working at outpatient department of the psychiatry clinic identified primary caregivers of mentally ill patients and referred them to data collectors. After that, the data collectors invited all eligible primary caregivers to participate in the study.

#### Severe mental illness

In this study, severe mental illness includes psychotic disorders (schizophrenia, schizoaffective, schizophreniform), depression and bipolar disorders. These diagnoses were obtained from patients’ charts.

### Instruments

All questionnaires were translated into local languages (Afan Oromo and Amharic) before data collection. Consistency was checked by a back-translation by another expert fluent both in English and in local languages.

#### Outcome variables

##### Depression

A structured questionnaire, patient health questionnaire (PHQ-9) was used to assess depression among primary caregivers. PHQ-9 score ranges from 0 to 27. Each of the 9 items were scored from 0 (“not at all”) to 3 (“nearly every day”). A PHQ-9 score 10–14 indicates moderate depression and 15–19 indicates moderately severe depression. And, a score of 20 to 27 on PHQ-9 indicates severe type of depression which requires immediate initiation of therapy [21, 22]. Moreover, PHQ-9 has been validated in Ethiopian healthcare context with specificity and sensitivity of 67% and 86% respectively. A cut-off point of 10 or more has been used to screen depression [23]. In the current study, Cronbach’s alpha of the scale was 0.90.

### Potential explanatory variables of depression

#### Demographic variables of primary caregivers

Age, sex, *religion*, ethnicity, monthly income, employment status, marital status, number of children, educational status.

#### Psychological factors

Perceived social support, perceived stigma of primary caregivers and the caregiver’s relation to the patient were among psychological factors considered in this study. Perceived social support was assessed by a multidimensional scale of perceived social support (MSPSS) scale which has 12 items. Among these 12 items, four are family related questions, four are friend related questions and the remaining four were related to significant others (people whom the individual values most). Each statement was rated by using a 5-point scale [24]. The MSPSS is a valid and reliable instrument to assess perceived social support in African context [25]. In this study, social support was treated as continuous variable and the Cronbach’s alpha of the scale was 0.95.

Perceptions of stigma related to mental illness was assessed using the family devaluation and stigma scale (FDSS) which had seven items that evaluate beliefs about the degree of devaluation and discrimination directed towards the families. Each statement was rated by using a 4-point scale, from 1(strongly disagree) to 4(strongly agree) with a total stigma score ranging from 0 to 28, and the higher scores indicating greater stigma [26]. We have treated stigma related to mental illness as continuous variable. In the current study, the Cronbach’s alpha of the scale was 0.86.

#### Alcohol use disorder (AUD)

AUD was assessed by using CAGE (Cut down Annoyed, Guilty Eye opener), which has 4 items. CAGE has been validated in Ethiopia [27]. A participant who scored two or more on CAGE was classified as having an alcohol use disorder.

#### Khat use

A self-reported questionnaire was used to assess khat use and frequency of khat use. In this study, current khat use was defined as using khat during the month prior to the interview.

#### Cigarette and cannabis

We have used a self-reported questionnaire to assess cigarette and cannabis smoking (current smoker/non-smoker and the number and frequency of cigarettes and cannabis smoked). Participants who smoked at least one cigarette or one stick of cannabis per day were considered as cigarette smokers or cannabis users.

#### Patients related factors

Types of diagnoses, previous suicidal attempts, duration of mental illness and history of hospital admissions were collected by reviewing charts of the patients.

#### Other factor

Duration of giving care to the patients.

### Data collection procedures

Data were collected by interviewing primary caregivers of patients with severe mental illness receiving services in outpatient department of psychiatry clinic at JUSH. Two psychiatric nurses and two postgraduate students in integrated community and clinical mental health (ICCMH) had participated in data collection.

Data collection was carried out after the questionnaires had been pretested on a sample (5% of the total sample) of primary caregivers of patients with severe mental illnesses receiving services at Jimma University Teaching Hospital. The pre-test results were not included in the final research report. Data collection was supervised by a bachelor degree level health officer. The supervisor monitored data quality and checked all questionnaires for completeness.

### Data processing and analysis

Data were entered into EpiDATA software package. After double data entry verification, data were exported from EpiData and analyzed using the Statistical Package for Social Science (SPSS, version 20). After data cleaning, association between dependent and independent variables was assessed using a bivariate logistic regression analysis, to estimate the strength of association using Odds Ratios (OR). Multivariate logistic regression was conducted to determine independent predictors of depression after controlling for confounders. All variables associated with depression with a p-value less than 0.25 in the bivariate logistic regression, were further analyzed using a multivariate logistic regression analyses to control for confounding. Variables with a p-value less than 0.05 were declared to be associated with depression.

### Ethical consideration

Ethical clearance was obtained from the ethical review board of Jimma University. Written informed consent was obtained from each of the participants prior to participation. Information obtained was kept confidential and anonymous during all stages of the study. Those who were identified to be severely depressed were linked to treating clinicians.

## Results

### Participants’ and patients’ characteristic

A total of 284 primary caregivers of psychiatric patients were invited to participate in the study. All the participants agreed to participate in the study with 100% of response rate. More than half (56.3%; *n* = 160) of the primary caregivers were females. Of the total primary caregivers, 35.6% (*n* = 101) and 20.4% (*n* = 58) were parents and children of the patients respectively. The mean age of primary caregivers was 37.73 ± SD 11.207 years, with ages ranging from 18 to 64 years. Around 30% (*n* = 83) of the primary caregivers were between age group of 35-44 years followed by 45-54 years (24.3%, *n* = 69). More than one-third of the primary caregivers could read and write (38%, *n* = 108). Nearly 57%, (*n* = 161) of the primary caregivers were from the Oromo ethnic group and nearly one-third of them (32%, *n* = 155) were Islam religion followers. Nearly two-third of the primary caregivers (63.2%, *n* = 179) were married. Most primary caregivers (88.4%, *n* = 251) were living together with the patients (See Table 1).

**Table 1:** Socio-demographic characteristics of primary caregivers of patients with severe mental illness in South West, Ethiopia (*n* = 284)

Characteristics		Frequency (%)
Sex	Male	124(43.7)
	Female	160(56.3)
Age of caregivers	18–24	39(13.7
	25–34	67(23.6)
	35–44	83(29.2)
	45–54	69(24.3)
	55–64	26(9.2)
Ethnicity	Oromo	161(56.7)
	Amhara	74(26.1)
	Keffa	17(6.0)
	Others	32(11.3)
Religion	Orthodox Christians	91(54.6)
	Muslim	155(32)
	Protestant	38(13.4)
Marital status	Single	64(22.5)
	Married	179(63.0)
	Divorced/ Widowed	41(14.4)
Level of education	Illiterate	65(22.9)
	read and write	108(38.0)
	primary (1–8)	58(20.4)
	secondary (9–12)	42(14.8)
	tertiary > 12	11(3.9)
Occupation of caregivers	Unemployed	27(9.5)
	Labor worker	20(7.0)
	Government employee	50(17.6)
	Farmer	74(26.1)
	Merchant	44(15.5)
	Housewife	42(14.8)
	Others	27(9.5)
Relationship to patients	Spouse	50(17.6)
	child	58(20.4)
	parent	101(35.6)
	relative	24(8.5)
	Sibling	44(15.5)
	others	7(2.5)
Frequency of attending a place of worship	Daily	149(52.5)
	2–3 times/week	107(37.7)
	never	28(9.9)
Living together with patients	Yes	251(88.4)
	No	33(11.6)

Other ethnicity: Guragie, Dawuro, Tigre, Silte, Somali

Other occupation: Students, being retired

Other relationship: Friends, paid caregivers

The mean age of the patients was 30.5+ SD 9.81 years, with ages ranging from 11 to 62 years. The highest percentage of patients (44%, *n* = 125) were between the ages of 18 to 27 years. The predominant diagnosis of the patients was schizophrenia 102(35.9%) followed by bipolar disorder (27.5%, *n* = 78) and depression (21.8%, *n* = 62).

### Prevalence of depression among primary caregivers of psychiatry patients

The overall prevalence of depression among primary caregivers of patients with mental illness was 19% (*n* = 54). Out of the total primary caregivers, 11.3% (*n* = 32), 3.5% (*n* = 10) and 4.2% (*n* = 12) of them had moderate, moderately severe and severe type of depression respectively. The prevalence of depression among female primary caregivers was 25% (*n* = 40). More than one-fifth (22.2%, *n* = 14) of the primary caregivers who had been giving care for more than four years were found to have depression. Of the primary caregivers who were using khat, 22.4% (*n* = 22) of them had depression. In addition, 27.95% (*n* = 31) of lifetime alcohol users were found to have depression. Nearly, one-fourth (23.2%, *n* = 96) of the primary caregivers who were giving care for patients between the age group of 18-27 years had depression. The prevalence of depression among primary caregivers of patients diagnosed with schizophrenia was 19.6% (*n* = 20). Similarly, the prevalence of depression among primary caregivers of patients who had more than two hospital admissions was highest compared to primary caregivers of patients who had no history of hospital admission (26.4% vs. 14.8%).

### Factors associated with primary caregivers’ depression in the bivariate analyses

In the bivariate analysis, depression among primary caregivers was negatively associated with male gender, attending secondary level of education, monthly income of more than 1987 Eth Birr, being patients’ sibling or relative, and social support. However, perceived stigma, patient’s having a single episode of suicidal attempt, life time alcohol use, spending more than six hours to give care to patients per day, never attending a place of worship and being a house wife were positively associated with primary caregivers’ depression.

### Factors predictive of primary caregivers’ depression

In the multivariable logistic regression model, having greater social support (aOR 0.94, 95% CI = 0.90, 0.98), having attended secondary school (aOR 0.09, 95% CI = 0.01, 0.64) and caring for a patient aged between 38 and 47 years (aOR 0.23, 95% CI = 1.05, 1.13) were negatively associated with depression among primary caregivers of patients with severe mental illness. On the other hand, being single (aOR 2.62, 95% CI = 1.07, 6.41), giving care more than six hours per day (aOR 3.75, 95% CI = 1.51, 9.33) and patient’s attempting suicide more than once (aOR 1.48, 95% CI = 1.07, 3.42) were positively associated with depression among primary caregivers of patients with severe mental illness. The odds of having depression among primary caregivers decreased by 6% for every one unit increment of perceived social support (aOR 0.94, 95% CI = 0.90, 0.98). The odds of depression among primary caregivers who had given care for more than six hours per day was nearly four times higher than that of caregivers who gave care for six hours and less (aOR 3.75, 95% CI = 1.51, 9.33). The odds of depression among primary caregivers of patients who had attempted suicide more than once was 1.48 times higher than that of caregivers of patients with no history of suicidal attempts (aOR 1.48, 95% CI = 1.07,3.42) (see Table 2). In the final model, gender, stigma, life time alcohol use, religion, ethnicity, occupation, monthly income, spending the whole week to give care, frequency of attending a place of worship, relationship to the patients and living together with the patient were not associated with depression among primary caregivers of patients with severe mental illness (see Table 2).

**Table 2:** Multivariate logistic regression of factors independently associated with depression among primary caregivers of patients with severe mental illness in south west, Ethiopia (*n* = 284)

Variables		*P*-value	AOR	95% C. I	
				Lower	Upper
Sex	Male	0.19	0.47	0.15	1.45
	Female		Reference		
Life time alcohol use	Yes	0.21	2.36	0.62	9.05
	No		Reference		
Marital status	Single	0.04	2.62	1.07	6.41
	Married		Reference		
	Divorced/widowed	0.85	1.13	0.33	3.90
Religion	Orthodox Christians	0.37	1.79	0.50	6.40
	Muslim		Reference		
	Protestant	0.19	2.77	0.61	12.51
Ethnicity	Oromo		Reference		
	Amhara	0.24	0.49	0.15	1.62
	Keffa	0.12	0.14	0.01	1.65
	Other	0.18	0.36	0.08	1.57
Occupation	Unemployed	0.18	0.28	0.04	1.83
	Labor worker	0.80	1.26	0.21	7.77
	Employee	0.94	1.07	0.19	6.02
	Farmer		Reference		
	Merchant	0.92	1.09	0.22	5.38
	Housewife	0.90	0.91	0.21	3.90
	Other	0.33	2.55	0.39	16.61
Income	<650 Eth birr		Reference		
	651-1000Eth birr	0.59	1.34	0.47	3.84
	1001–1897 Eth birr	0.90	1.08	0.31	3.83
	>1987Eth birr	0.07	0.28	0.07	1.08
Hours spent by caregiver per day to give care	Six hours and less		Reference		
	More than six hours	0.004	3.75	1.51	9.33
Suicidal attempts	No attempt		Reference		
	Only once	0.52	1.39	0.51	3.80
	Twice and more	0.04	1.48	1.07	3.42
Age of the patients	18–27		Reference		
	28–37	0.10	0.46	0.18	1.16
	38–47	0.01	0.23	1.05	1.13
	48–57	0.24	2.49	0.54	11.43
Frequency of attending a place of worship	Daily		Reference		
	2–3 time/week	0.23	1.74	0.71	4.27
	Never	0.57	1.50	0.37	6.06
Level of Education	Illiterate		Reference		
	Read and write	0.08	0.38	0.13	1.11
	Primary	0.09	0.34	0.10	1.19
	Secondary	0.02	0.09	0.01	0.64
	Tertiary	0.36	0.28	0.02	4.20
Relationship to Patients	Spouse	0.64	1.31	0.42	4.13
	Child	0.63	1.36	0.39	4.82
	Parents		Reference		
	Siblings/relative	0.08	0.24	0.05	1.17
Living together with patient	Yes	0.69	1.45	0.24	8.63
	No		Reference		
Perceived social support		0.001	0.94	0.90	0.98
Perceived stigma		0.08	1.10	0.99	1.22

## Discussion

In this cross-sectional survey of primary caregivers of patients with mental illness in Southwest Ethiopia, nearly one-fifth (19%) had depression.

The prevalence of depression found in this study (19%) was comparable to the findings of the study conducted in Buraydah Mental Health Hospital, Saudi Arabia (23.33%) [11] among those with schizophrenic disorders that used DSM IV-TR criteria to assess depression.

However, the prevalence of depression found in this study was lower than the findings of a study conducted in Egypt among female caregivers in psychiatry clinic that used DSM IV-TR criteria to assess depression (34%) [28]. This discrepancy may be due to difference in the characteristics of study participants in terms of gender. In addition, caregivers of patients with mental and physical illness were included in the study conducted in Egypt [28]. Similarly, the prevalence of depression in our study (19%) was lower than the prevalence reported from India (27.5%) [15], Sri Lanka (37.5%) [29], California USA (40%) [10], and Rhode Island USA (75%) [9]. The inconsistency between our findings and that of these four studies may be due to the difference between screening tools. While we used PHQ-9 in our study, studies conducted in California and Sri Lanka used the CES-D; and the Indian study used the MADRS. Moreover, there is strong social cohesion in Ethiopia which is a good source of social support though further investigation is needed to determine its effect on the prevalence of depression.

In our study, the prevalence of depression among female primary caregivers was higher than that of male primary caregivers (25% versus 11.3%, *p* = 0.004). On the other hand, even though, the prevalence of depression among female primary caregivers in our study was higher than that of the male counterparts, it was still lower than the findings of Egyptian study (34%) [28]. Moreover, in the current study, 26.3% of females were housewives, who are expected to spend more of their time in care giving at home. The prevalence of depression among primary caregivers who were housewives was higher than that of farmer primary caregivers (31% vs 13.5%, *p* = 0.03). In Ethiopian culture, most household chores are handled by housewives. So, the double burden of household chores and caring for the patients might have put women at more risk of developing depression. In addition, as housewives spend most of their time at home, they have prolonged contact with the patients.

The current study indicated that the prevalence of depression among primary caregivers who had never attended a place of worship was higher than that of primary caregivers who had attended a place of worship daily (35.7% vs15.4%, *p* = 0.01). One possible explanation for this difference may be the fact that primary caregivers who had attended a place of worship daily received emotional support from religious leaders. Primary caregivers may also receive social support at their places of worship. The effect of social support on depression among caregivers was reported in the study done in the USA [20].

In the current study, giving care for more than six hours was associated with depression among primary caregivers which agrees with the study done in Saudi Arabia [11]. Having ever attended secondary school education was negatively associated with depression among primary caregivers which agrees with the study conducted in California [10]. However, giving care for patients who had two or more episodes of suicidal attempts was positively associated with depression among primary caregivers. The odds of having depression among primary caregivers increased by 16% with a unit increment in perceived stigma in the bivariate analysis. The possible reason may be because depression has strong association with perceived stigma among caregivers of patients with mental illness as reported in other study [30].

In the multivariate logistic regression, low perceived social support, being single, giving care for more than six hours, giving care for a patient with a history of suicidal attempts and illiteracy were positively associated with depression among primary caregiver of patients with mental illness.

Since we used a cross-sectional study design, it is difficult to establish a cause-effect relationship. In addition, the tools we used to assess social support and stigma were not validated in the Ethiopian context. Nevertheless, the findings will help to inform practice and future research, specifically in Ethiopia and other developing countries.

## Conclusion

In this study, the prevalence of depression among primary caregivers was high. This may have its own impact on the treatment outcome and prognosis of patients with mental illness. In addition, the prevalence of depression among female caregivers was higher than that of the male caregivers. Depression among primary caregivers was associated with providing care for an increased number of hours per day; low perceived social support and the patient’s suicidal attempts. Therefore, while caring for patients, screening primary caregivers of patients with mental illness and treating them accordingly, is crucial to decrease the incidence of depression. Special focus should be given to primary caregivers spending long hours for providing care, those with low perceived social support; caregivers of patients with suicidal ideation and female primary care givers.
